# VARIATION IN ASSISTED LIVING REGULATION: IMPLICATIONS FOR STATE AGENCY OVERSIGHT, PROVIDER KNOWLEDGE, AND PRACTICE

**DOI:** 10.1093/geroni/igad104.0209

**Published:** 2023-12-21

**Authors:** Lindsey Smith, Derek Manis

## Abstract

Researchers spend significant time and effort documenting policy, particularly in assisted living, where there are no national regulatory standards, and state governments develop and enforce regulations, leading to significant variation between and within states. Our panel explores the question we often get—do these regulations matter? Do they really impact the structures, processes, and outcomes of care provision?

